# Supplementary material for: A high throughput screen for active human transposable elements
Source: BMC Genomics. 2018 Feb 1;19:115. doi: 10.1186/s12864-018-4485-4 (PMC5796560; doi:10.1186/s12864-018-4485-4)
Supplement: Additional file 1: — Supplemental Material. Contains detailed procedures, all supplemental figures (Figure S1-S6), and supplemental tables (Tables S1-S4). (PDF 1569 kb) [file 12864_2018_4485_MOESM1_ESM.pdf]

Supplemental Material: A high throughput screen for active human transposable elements

Erika M. Kvikstad[1,2], Paolo Piazza[1,3], Jenny C. Taylor[1,2], Gerton Lunter[1]

[1]Wellcome Trust Centre for Human Genetics, Oxford, UK
[2]National Institute for Health Research Comprehensive Biomedical Research Centre, Oxford, UK
[3]Department of Medicine, Imperial College London, London, UK

DETAILED PROCEDURES

Materials

DNA quantity and quality

Genomic DNA libraries can be prepared from as little as ~100ng of genomic DNA. Quantify the samples using a fluorescence-incorporation approach to ensure accuracy (eg, using Nanodrop, Qubit spectrophotometer or, for larger batches, picogreen is preferable). DNA sample integrity and median library size can be determined using Agilent 2200 Tapestation and Genomic screen tape.

Reagents

Ensure that sufficient volumes of the following reagents are available:

- NEBNext Ultra DNA Library Kit for Illumina (New England Biolabs, cat. no.E7370)
- RecJf (New England Biolabs, cat. no.M0264L)
- NEBNext Q5 High-fidelity 2x PCR master mix (New England Biolabs, cat. no.M0541L)
- Magnesium chloride $Mg2^{+}$ (25mM; VWR, cat.no.PAA3513)
- DNase/RNase-free H20 ultraPure (Life Technologies Ltd, cat. no. 10977-015)
- Dual index primers 25 μM (Table S2)
- TE protocol primers 25 μM (Table S2)
- 80% (v/v) ethanol
- Agencourt AMPure XP magnetic beads (Beckman Coulter, cat. no.A63882)
- EB elution buffer (Quiagen Ltd, cat. no. 19086)
- Qubit Molecular probes dsDNA HS assay kit (Life Technologies Ltd, cat. no. Q32854)
- Agilent Tapestation D1000 reagents (Agilent Technologies, cat. no. 5067-5583)
- Agilent Tapestation D1000 Screen Tape (Agilent Technologies, cat. no. 5067-5582)
- Agilent Tapestation High sensitivity D1000 reagents (Agilent Technologies, cat. no. 5067-5585)
- Agilent Tapestation High Sensivity D1000 Screen Tape (Agilent Technologies, cat. no. 5067-5584)
- Agilent Tapestation Genomic DNA Reagents (Agilent Technologies, cat. no. 5067-5366)
- Agilent Tapestation Genomic DNA Screen Tape (Agilent Technologies, cat. no. 5067-5365)

Equipment

- Thermal cycler, e.g. DNA Engine tetrad PTC-225 (MJ Research, Bio-Rad), or equivalent
- Microcentrifuge
- Qubit 2.0 fluorometer (Life Technologies Ltd, cat. no. Q32866)
- Qubit assay tubes (Life Technologies Ltd, cat. no. Q32856)

- Agilent 2200 Tapestation (Agilent Technologies)
- Benchtop vortex, e.g. (VWR, cat. no. 82019-170), or equivalent
- Centrifuge with 96-well SBS plate carrier (e.g. Beckman), or equivalent
- DynaMag-96 side skirted magnet (Life Technologies Ltd, cat.no.12027)
- Filtered pipette tips, e.g. Rainin P20, P200 (Anachem Ltd, cat. no.s RTL10FLR, RLT200F), or equivalent
- Agilent Tapestation loading tips (Agilent Technologies, cat. no. 5064-5153)
- Optical Tube strips (Agilent Technologies, cat. no. 401428)
- Optical Strip caps (Agilent Technologies, cat. no. 401425)
- 96-well SBS plate, skirted, 0.2 mL capacity, e.g. Eppendorf twin.tec 96-well PCR plates, fully-skirted (VWR, cat. no. 47744-116), or equivalent
- Test tube 1.5mL LoBind PCR (Eppendorf, cat. no. 80077-230), or equivalent

Procedures

Preparation of genomic libraries

Prepare genomic libraries for next generation whole genome sequencing according to NEBNext Ultra DNA Library Kit (New England Biolabs, Ipswich, Massachusetts) for Illumina which performs well with low input genomic DNA (~100 ng) used here.

NB: if starting with existing whole genome libraries, proceed to step 4.

1. Fragment genomic DNA to desired size range (median 500 bp) using sonication (e.g., Covaris, Woburn, Massachussets), or enzymatic digestion (e.g., NEBNext dsDNA Fragmentase, cat. no. M0348) according to manufacturer's specifications.
2. Proceed with end repair and adaptor ligation as per manufacturers specifications.
3. Generate library fragments with full length adapters by PCR extension using index primers (Table S2) and following manufacturer's specifications.

Initial DNA purification and size selection of library

Perform a solid phase reversible immobilization (SPRI) size selection and DNA purification to retain DNA fragments larger than ~ 200 bp, using Agencourt AMPure XP magnetic beads (Beckman Coulter, Brea, California) and a ratio **0.85x** DNA library volume.

NB: If generating library in steps 1-3 above, proceed to step 15.

4. Increase volume of DNA library to 50 μL using EB buffer
5. Allow AMPure Xp beads to equilibrate at room temperature. Gently shake the AMPure XP bottle to resuspend the magnetic beads.
6. Add **0.85x** ratio beads to DNA library volume: 42.5 μL beads per 50 μL DNA library. Mix thoroughly by pipetting up and down 15-20 times until the solution appears homogenous. (NB: do not increase the volume to 1:1 ratio; a less-than-standard ratio ensures that adapter dimer carryover is minimized.)
7. Incubate at room temperature for ~5 minutes. This step allows binding of PCR products approximately 150 bp and larger to bind the magnetic beads, and ensures maximum recovery.

8. Transfer the plate to an Agencourt DynaMag-96 side skirted magnet bar plate, and allow to sit for 5-10 minutes or until the solution is clear and beads formed in a pellet.
9. Slowly aspirate the cleared solution from the reaction plate, while avoid perturbing the bead pellet. Retain the supernatant until confirmation of maximum recovery by quantification/sizing.
10. Dispense 150 μL of 80% ethanol solution to each well and incubate for 30 seconds at room temperature. Aspirate and discard. Repeat for a total of 2 ethanol washes.
11. Place the reaction plate on bench top to dry for ~5 minutes.

NB: Be sure to remove all ethanol from the bottom of the well, as this may contain residual contaminants. Allow ethanol to evaporate completely (bead pellet will appear opaque, no longer reflective), but avoiding over-drying of pellet (cracking) as this increases the difficulty of fluid resuspension.

12. Elute DNA from SPRI beads in 20 μL of EB elution buffer (dih20, TRIS-Acetate, or TE), seal to vortex for 30 seconds or pipette mix 10 times.

13. Incubate at room temperature for 2 minutes for efficient elution.
14. Place on magnet for ~1-2 minutes to allow beads to separate from eluted DNA, and transfer the eluate to a clean PCR reaction tube/plate.

Post library prep quality control

15. Quantify the library concentration using Qubit (Life Technologies, Carlsbad, California) according to the manufacturer's instructions. A minimum of 10 ng is desired for further processing.
16. Determine size distribution of library fragments using Agilent Tapestation, and High-Sensitivity screen tape (Agilent Technologies, Santa Clara, California). An expected library profile should be free of short (<100 bp) fragments and display a peak at ~500 bp -1 kb (Fig. S1).

Normalization

17. To achieve approximately uniform 10ng of post-PCR library for each input sample, manually add appropriate volume of DNA determined by quantification in step 15 to equal ~10 ng of sample for subsequent PCR reactions.

Target amplification

To exponentially amplify in multiplex fragments containing 3' sequence and flanking sequence of L1 and Alu insertions.

18. Assemble the following PCR reaction per sample on ice as follows, then vortex gently and centrifuge the solution:

Component	Volume (μL)	Final Concentration
DNA library (10ng)	varies	-
$dH_2 0$	to 17 μL	-
NEB Q5 High fidelity 2x PCR mastermix	25	1x
Mg2+ (25 mM)	4	2 mM
Alu-target primer (25 μM)	1	0.5 μM
L1HS-target primer (25 μM)	1	0.5 μM
Illumina Universal PCR primer (25 μM)	2	1 μM
total	50	-

19. Carry out the PCR reaction using the following thermal cycling parameters:

Cycles	Temperature (C)	Time	Step
1	98	2 min	Denaturation
	98	10 sec	Denaturation
10	67	30 sec	Annealing
	72	30 sec	Extension
1	72	5 min	Final extension
1	10	forever	Hold

Post-PCR Purification

20. Repeat steps in 4-14, using 90 μL of AMPure XP beads per 50 μL of PCR reaction volume in a **1.8 x** ratio. Elute in 20 μL EB elution buffer. This will minimize the carryover of of Illumina Universal PCR primer primer, and selectively retain fragments approximately >100 bp.

P1 asymmetric amplification

To asymmetrically (linearly) amplify fragments containing sequence complimentary to TE-targeting primers. Double-stranded DNA (dsDNA) products include linear amplification from TE targets, and exponential amplification of TE targets oriented in head-to-head fashion. Background genomic DNA molecules lacking TE priming sites remain largely denatured (due to high complexity), in single- stranded DNA (ssDNA) form.

21. Assemble the following PCR reaction mix on ice, followed by gentle vortex mixing and centrifugation:

Component	Volume (μL)	Final Concentration
Clean PCR step 20	19	-
dH20	NA	-
NEB Q5 High fidelity 2x PCR mastermix	25	1x
Mg2+ (25 mM)	4	2 mM
Alu-target primer (25 μM)	1	0.5 μM
L1HS-target primer (25 μM)	1	0.5 μM
total	50	-

22. Carry out the PCR using the following parameters:

Cycles	Temperature (C)	Time	Step
1	98	2 min	Denaturation
2	98	10 sec	Denaturation
	67	30 sec	Annealing
	72	30 sec	Extension
1	10	forever	Hold

Digestion of background ssDNA fragments

Perform ssDNA exonuclease digestion to remove unwanted ssDNA fragments from the library.

23. Assemble the RecJf exonuclease reaction (total volume 60 μL) reaction per sample on ice as follows, then vortex gently and centrifuge the solution:

Component	Volume (μL)
PCR reaction	50
dH20	3
NEBuffer 2 (10x)	6
RecJf	1
total	60

24. Incubate the reaction according to the following parameters:

Step	Cycles	Temperature (C)	Time
Digestion	1	37	30 min
Inactivation	1	65	20 min
Hold	1	10	forever

Post-digest DNA Purification

25. Repeat steps 4-14 to remove salts, buffer, enzyme, and primer 1 from solution, and perform size selection to retain fragments > approx. 100 bp. Adding **1.8 x** ratio beads to reaction volume: 108 μL beads per 60 μL RecJf reaction volume. Elute in 20 μL EB elution buffer.

P2 asymmetric amplification

To asymmetrically amplify fragments containing sequence complimentary to the Illumina universal adaptor sequence. dsDNA products at this stage include linear amplification from TE targets, and remaining background genomic fragments undigested in steps 23-24. Molecules containing TEs in head-to-head orientation lacking Illumina Universal (P5) PCR primer priming sites remain largely denatured (due to high complexity), in ssDNA form, for subsequent removal by exonuclease digestion.

26. Assemble the following PCR reaction mix on ice, followed by gentle vortex mixing and centrifugation:

Component	Volume (μL)	Final Concentration
Clean PCR	20	-
dH20	4	-
PCR mastermix (2x)	25	1x
Illumina Universal PCR primer (25 μM)	1	0.5 μM
total	50	-

27. Carry out the PCR using the following thermal cycling conditions:

Cycles	Temperature (C)	Time	Step
1	98	2 min	Denaturation
2	98	10 sec	Denaturation
	63	30 sec	Annealing
	72	30 sec	Extension
1	10	forever	Hold

Digestion off-target ssDNA fragments

28. Repeat steps 23-24 to perform ssDNA exonuclease digestion to remove unwanted ssDNA fragments from the library.

Nested amplification

Perform nested amplification with a primer designed for further TE specificity, and containing nucleotides to partially reintroduce the Illumina index adaptor. One primer is designed per element (L1HS, AluYa5/8, and AluYb8/9, respectively). Use a dilution of library digested in step 28 as template DNA.

29. Assemble 3 PCR reaction mixes on ice, one per nested primer, followed by vortex mixing and centrifugation:

**Component**	**Volume (μL)**
Digested library step 28	4
dH20	19
Q5 High Fideliy 2x PCR mastermix	25
Nested primer (25 μM)	1
Illumina Universal P5 PCR primer (25 μM)	1
total	50

30. Carry out each PCR amplification according to the following thermal cycling parameters, using the optimal Ta per nested primer (below):

**Cycles**	**Temperature (C)**	**Time**	**Step**
1	98	2 min	Denaturation
15	98	10 sec	Denaturation
	Ta	30 sec	Annealing
	72	30 sec	Extension
1	72	5 min	Final extension
1	10	forever	Hold

**Primer**	**Ta**
L1HS-nested primer	68
AluYa5/8-nested primer	68
AluYb8/9-nested primer	64

Post-PCR Purification

31. To remove salts, buffer, enzyme, and primers from solution, and perform size selection to retain fragments > approx. 100 bp. Follow steps in 4-14, adding **1.8 x** ratio beads to reaction volume: add 90 μL beads per 50 μL PCR reaction volume and elute in 20 μL EB buffer.

Adapter extension

Generate full-length double-stranded libraries with two adaptors compatible with Illumina bridge sequencing chemistry.

32. Assemble the following PCR reaction mix on ice, followed by gentle vortexing and centrifugation:

**Component**	**Volume (μL)**
Clean PCR step 31	18
dH20	NA
PCR mastermix (2x)	25
Illumina Universal P5 PCR primer (25 μM)	1
Index PCR primer 1 P7 (25 μM)	1
total	5

33. Perform PCR according to the following thermal cycling conditions:

**Cycles**	**Temperature (C)**	**Time**	**Step**
1	98	2 min	Denaturation
5	98	10 sec	Denaturation
	65	30 sec	Annealing
	72	30 sec	Extension
1	72	5 min	Final extension
1	10	forever	Hold

Final clean-up

34. To remove remaining primers, enzyme, incomplete adapter extension fragments, and adaptor-adaptor fragments. Follow steps outlined in 4-14, adding **1x** ratio AMPure XP beads to reaction volume to retain fragments ~150 bp and larger. Elute in 20 μL EB buffer.

Library pooling for sequencing

Prepare a pool of libraries originating from each sample, and for each TE element, as follows:

35. Calculate the molarity of each library by obtaining the concentration (eg, Qubit) and size distribution (Agilent Tapestation or Bioanlyzer). A typical library should be free of short (<100 bp) fragments and display a peak at ~300 bp, i.e. half the size of the starting library (Fig. S1)
36. Create a pool of libraries according to the ratios below:

TE	Fraction of pool	Expected reads/sample
L1HS	0.14	0.1*10^6
AluYa5/8	0.72	0.5*10^6
AluYb8/9	0.16	0.11*10^6

NB: The relative quantities of each sample were determined using conservative values of a mean coverage of 50x per TE locus, to achieve a minimum of 5 reads for >= 95% loci.

37. Determine the concentration and size distribution of fragments in the pool as above.
38. Prepare a dilution of the library pool, aiming for a final 10 nM solution in a final concentration of 0.1% tween, ready for Illumina sequencing.

DNA sequencing

39. Perform paired end sequencing of the pooled libraries on Illumina Miseq, HiSeq 2000, or 2500 instrument according to the manufacturer's specifications (Illumina Inc, San Diego CA).

SUPPLEMENTARY FIGURE LEGENDS

Fig. S1. Example TE-enriched library fragment distributions. Electropherograms of TE-enriched libraries analyzed by Agilent Tapestation D1000 Screen Tape. Profiles of NA12878 libraries produced following the described method are representative for (A) L1HS, (B) AluYa5/8, and (C) AluYb8/9-enriched libraries.

Fig.S2. Computational pipeline for TE detection from targeted sequencing. Schematic of the data analysis pipeline. First, reads are filtered and aligned to the human reference genome (hg19). Reads are filtered for features characteristic of the TE-targeted sequencing scheme, namely: (i) presence of primer and (ii) TE-like sequence on Illumina read 2; (iii) mapping quality of unique flanking sequence generated from Illumina read 1. Next, alignments are clustered by genomic position. Last, clusters are annotated against a comprehensive local database compiled from publicly available TE insertion profiles (polyTEdb; see Materials and Methods for details).

Fig. S3. Concordance among NA12878 TE calls generated by various detection methods. Venn diagram illustrating the intersection of TE calls (within 100 bp overlap) among (A) LINE1 and (B) Alu observed in NA12878. MEI, Mobile Element Insertion [1]; Phase 3, 1000 Genomes Phase 3 [2]; Ewing, L1 detection [3]

Fig. S4. Example NA12878-specific 1000 Genomes Phase 3 AluYb9 insertion observed by TE-enriched sequencing. IGV screenshot of an AluYb9 insertion observed in TE-enriched sequencing libraries generated from NA12878 sample and supported by Oxford Nanopore Technology (ONT) evidence. Two types of ONT evidence were used to manually verify novel TE insertions in NA12878, as illustrated for a known polymorphic insertion observed in 1000 Genomes Phase 3 MEI calls. (A) Hard clipping of reads spanning junction between flanking sequence and 3' end of TE. (B) Multiple insertion "pile-up" at junction corresponding to inserted TE sequence absent from hg19. Top panel, RepeatMasker annotations in hg19 genome reference. 1st bam, NA12878 WGS produced by ONT; 2nd bam, NA12878 L1HS-enriched library; 3rd bam, NA12878 AluYa5/8-enriched library; 4th bam, NA12878 AluYb8/9-enriched library.

Fig. S5. Novel L1HS insertion absent from reference genome and polyTEdb annotations. (A) IGV screenshot of alignments spanning novel L1HS locus inherited in trio A. Locus junction is spanned by Illumina read 1 reads in proband (WTCHG_180287_225.bam), mother (WTCHG_180287_226.bam), and father (WTCHG_180287_227.bam). IGV colors denote paired reads mapping to discordant chromosomes: chr8 (dark pink/purple), chr10 (pink). (B) Corresponding Illumina read 2 reads from discordant paired ends of fragments spanning a novel L1HS insertion in panel A. Blue bar represents a reference (hg19) annotated full-length L1HS on chr8. The full-length reference L1HS is detected in the assay by concordant read pairs; additional Illumina read 2 reads lacking sufficient sequence to span the unique flanks of novel L1HS will map to highly identical, full-length L1HS in the reference such as this locus.

Fig. S6. Distribution of mismatches to TE-like sequences in filtered reads derived from clusters. Example AluYb8/9 (A) Density (proportion of sequences) with a mismatch to the consensus sequence for each position in the TE 3' end. From left to right, position 0 corresponds to the 5' most TE-nested primer, to last nucleotide of TE-like sequence (excluding 3' poly-A tai). (B) Histogram of number of positions with a nucleotide mismatch vs. number of sequences observed with that number of mismatches. Reference True Positives (TP), NA12878 TP, False Positives (FP).

SUPPLEMENTAL TABLES

Table S1. Target and off-target control TE subfamilies in the human reference genome.

	TE	Copies[a]	Length[c] (10^6 bp)
Target	L1HS	1506	3.29
Target	AluYa5/8	4175	1.17
Target	AluYb8/9	3138	0.88
Off-target	L1PA2	4805	9.41
Off-target	AluSx1	109589	31.87

[a]Copies, number of loci present in the human reference (hg19) as annotated by RepeatMasker and excluding chrY

[c]Length, total genome length calculated from hg19 annotations (excluding chrY)

Table S2. Oligonucleotides for TE-enriched library construction.

Oligonucleotide	Reference	Sequence
Index PCR primer 1 (i7/P7)	[4]	5'-CAAGCAGAAGACGGCATACGAGAT-[INDEX[1]]-GTGACTGGAGTTCAGACGTGTGCTCTTCCGATC*T-3'
Universal PCR primer (i5/P5)	[4]	5'-AATGATACGGCGACCACCGAGATCTACAC TCTTTCCCTACACGACGCTCTTCCGATC*T-3'
L1HS-target primer	[5]	5'-GGGAGATATACCTAATGCTAGATGACAC-3'
Alu-target primer	NA	5'-AGCTTGCAGTGAGCCGA-3'
L1HS-nested primer	NA	5'-AGACGTGTGCTCTTCCGATCTTGCAC AATGTGCACATGTACCCTAAAACTTAG-3'
AluYa5/8-nested primer	NA	5'-AGACGTGTGCTCTTCCGATCTCGAGA TCCCGCCACTG-3'
AluYb8/9-nested primer	NA	5'-AGACGTGTGCTCTTCCGATCTGCAGTCCG CAGTCCG-3'

[1]Index, one of 96 unique 8-mer barcodes
NA, primers developed in TE-NGS

Table S3. TE-enriched sequencing reads derived from NA12878 NGS libraries

TE Library	Total Reads	Background[a] (%)	Filtered[b]	Clusters[c]	Reference Off-target[d]	Reference target[e]
L1HS	334066	28618 (0.078)	127538	4512	705 (16026)	663 (93826)
AluYa5/8	553933	28489 (0.051)	486810	62863	9586 (70896)	2606 (53716)
AluYb8/9	222967	29189 (0.13)	87525	6293	574 (3988)	2029 (67940)

[a]Background, reads defined as Illumina read 2 lacking discernable primer sequence in first 10 cycles of synthesis
[b]Filtered, reads mapped to hg19 and from filtered read pairs having Illumina read 2 matching exact primer sequence and containing matches to TE-like sequence, and Illumina read 1 with minimum mapping quality (see Materials and Methods for details)
[c]Clusters, genomic windows containing filtered reads grouped by hg19 coordinate position and containing minimum of 1 Illumina read 1
[d]Off-target, loci (reads) observed with minimum 1 Illumina read 1 mapping to hg19 annotated off-target controls (L1PA2 and AluSx1 for L1HS and AluYa58/AluYb89, respectively)
[e]Reference target, hg19 TE loci (reads) observed with minimum 1 Illumina read 1 mapping to predicted TE subfamily

Table S4. TE-enriched sequence reads and clusters generated for 12 ClinEx genome samples

		L1HS					AluYa5/8					AluYb8/9				
Sample	State[a]	Reads Total	Clusters	Ref TP[b]	Known Nonref[c]	Novel[d]	Reads Total	Clusters	Ref TP	Known Nonref	Novel	Reads Total	Clusters	Ref TP	Known Nonref	Novel
1	P	305548	1150	628	313	11	1802106	46433	2554	1112	29	329396	2412	1715	490	3
2	M	260698	1084	614	312	14	1789088	47251	2553	1128	30	319147	2523	1748	526	1
3	F	248874	1114	636	309	13	1041334	30214	2381	738	9	336392	2348	1671	447	6
4	P	372868	1481	627	321	89	1989809	48644	2587	1143	242	380150	2589	1729	517	33
5	M	322080	1282	619	305	61	1869648	48099	2580	1140	249	359665	2701	1747	552	29
6	F	415449	1600	632	336	88	1833514	46821	2552	1137	225	425731	2971	1770	588	33
7	P	358355	1489	649	343	77	1729632	45181	2584	1154	88	803936	3481	1781	623	45
8	P	197413	269	10	6	108	1074511	9	1	0	1	413993	0	0	0	0
9	P	275143	1340	634	321	45	772839	28328	2527	871	31	175274	2333	1705	474	5
10	P	226176	1132	630	337	17	727861	24407	2335	670	8	184436	2094	1624	397	1
11	P	391069	1514	648	380	45	725361	25132	2396	699	28	193735	2200	1644	423	6
12	P	265181	1219	642	324	23	767638	24647	2402	684	10	244582	2227	1657	408	2

[a]State, affected status of sample: P, proband; M, mother; F, father
[b]Ref TP, observed reference true positive insertions with a TE cluster within 600 bp window of 3' terminal position and match to predicted TE subfamily
[c]Known Nonref, known non-reference insertions with a TE cluster mapping within 600 bp window of 3' terminal position (if strand orientation provided)
[d]Novel, loci lacking evidence of TE insertion within 600 bp window of cluster position.

Fig. S1

A.

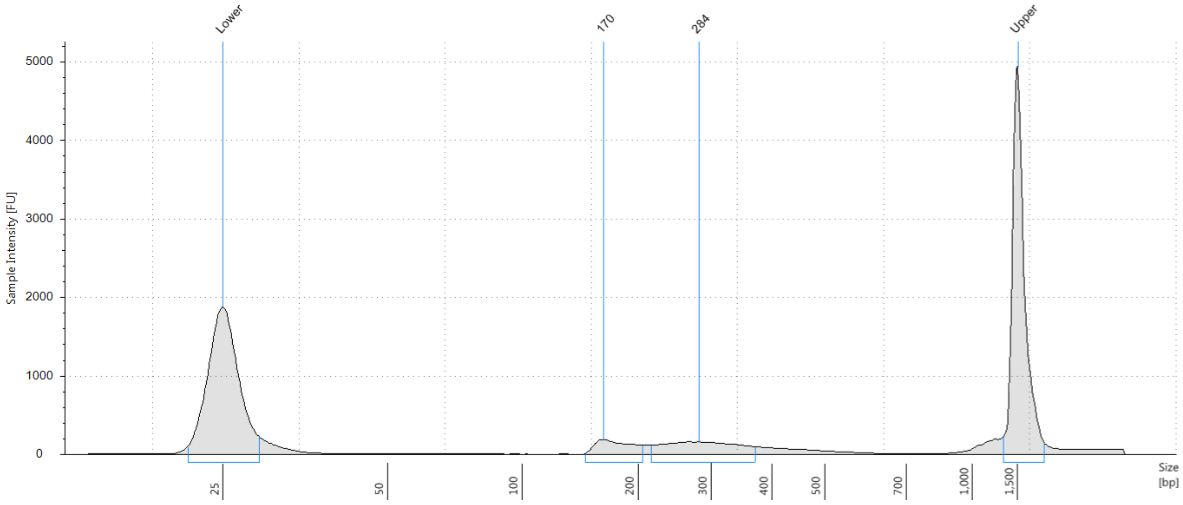
Lower
170
284
Upper
Sample Intensity [FU]
5000
4000
3000
2000
1000
0
25
50
100
200
300
400
500
700
1,000
1,500
Size [bp]

B.

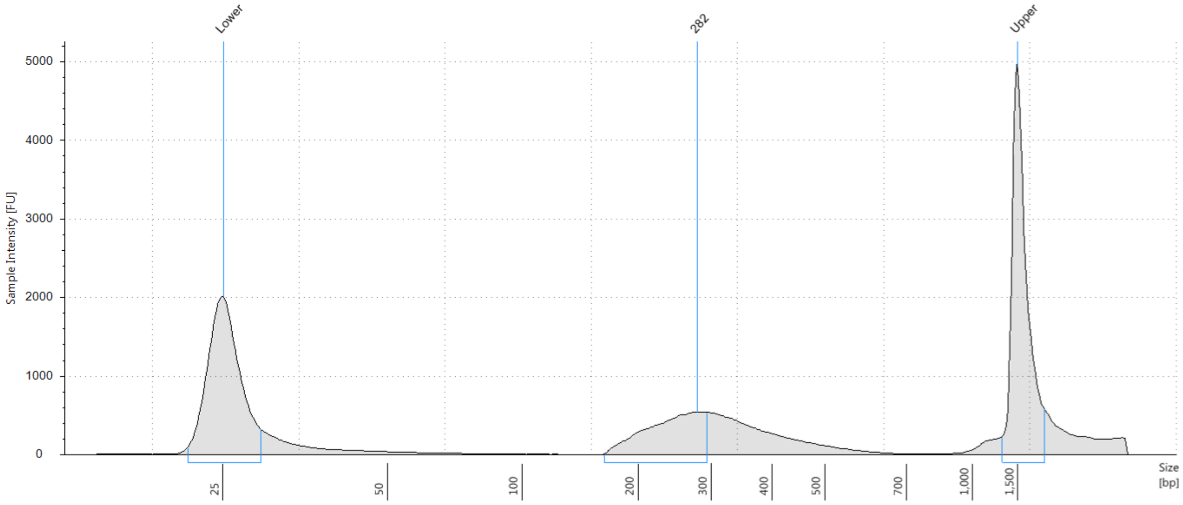
Lower
282
Upper
Sample Intensity [FU]
5000
4000
3000
2000
1000
0
25
50
100
200
300
400
500
700
1,000
1,500
Size [bp]

C.

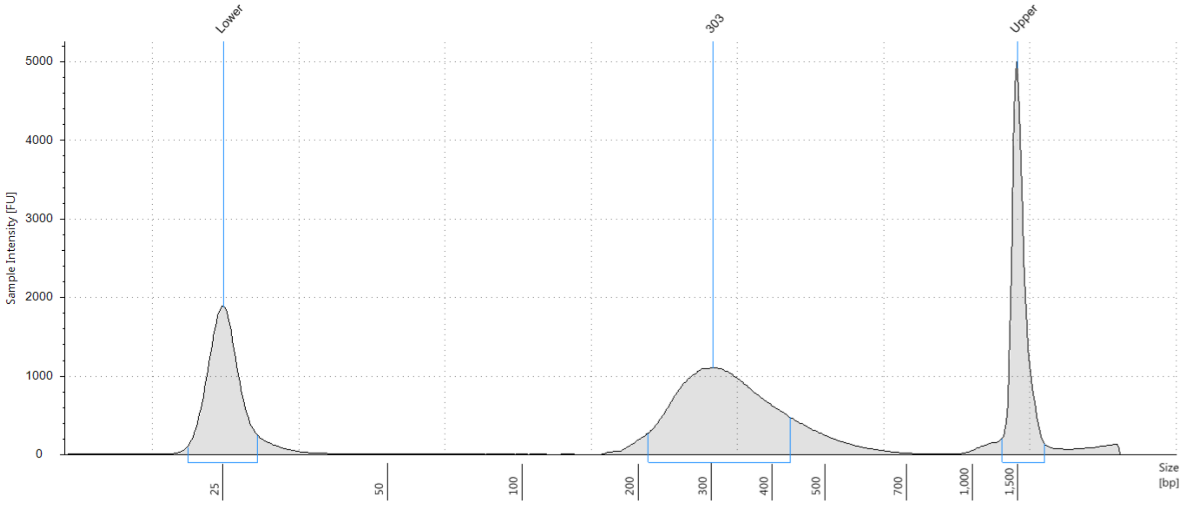
Lower
303
Upper
Sample Intensity [FU]
5000
4000
3000
2000
1000
0
25
50
100
200
300
400
500
700
1,000
1,500
Size [bp]

Fig. S2

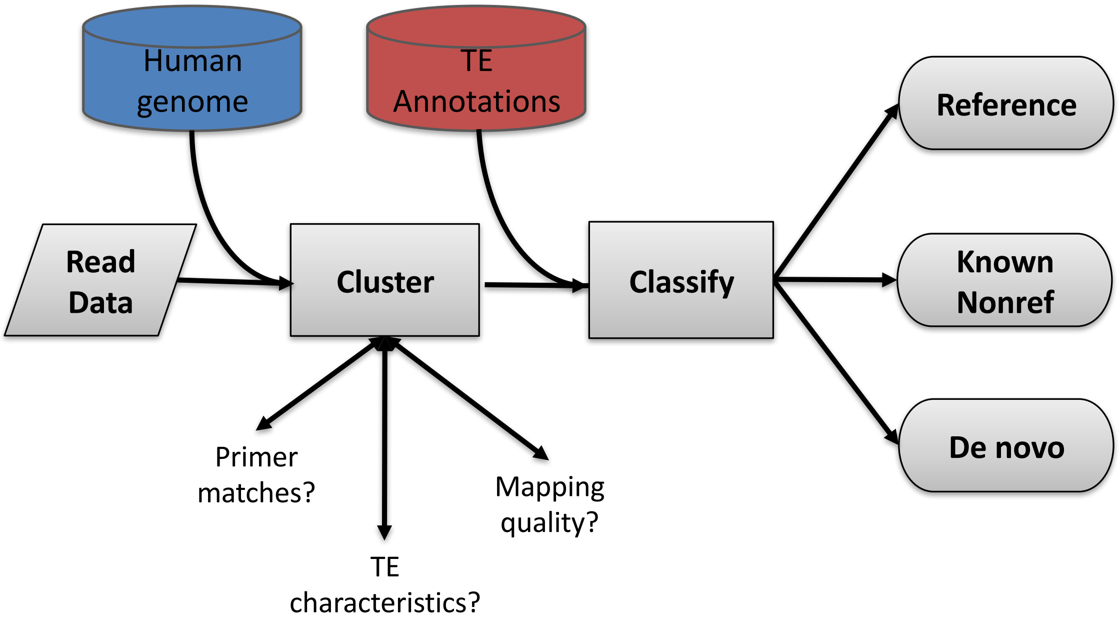
Human
genome
TE
Annotations
Reference
Read
Data
Cluster
Classify
Known
Nonref
De novo
Primer
matches?
Mapping
quality?
TE
characteristics?

Fig. S3

A.

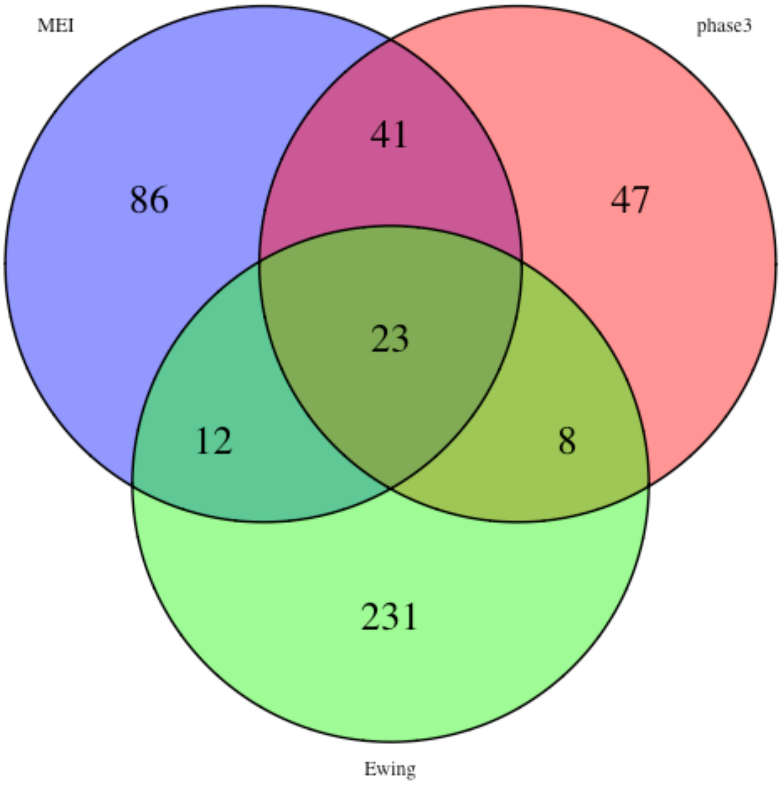

B.

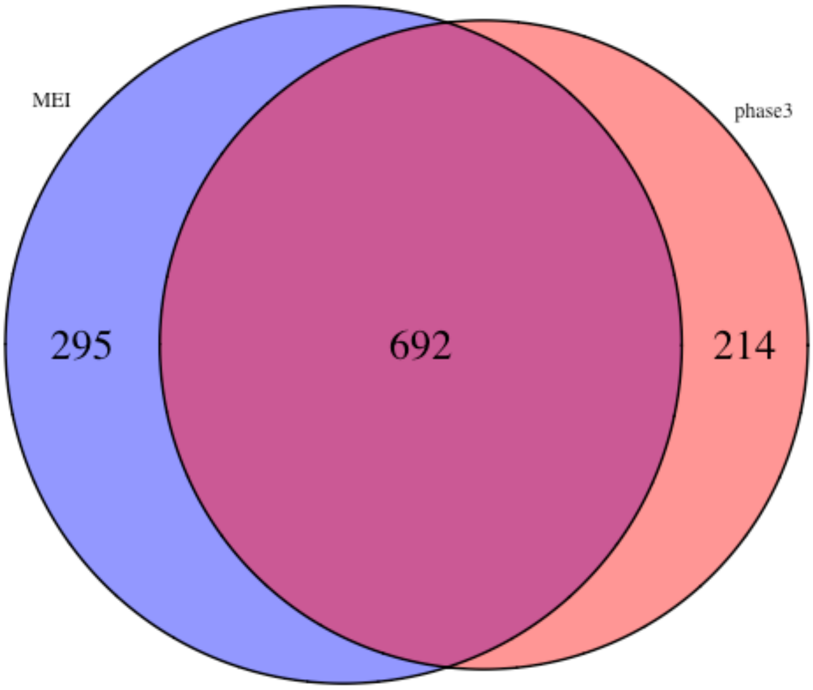

Fig. S4

A.

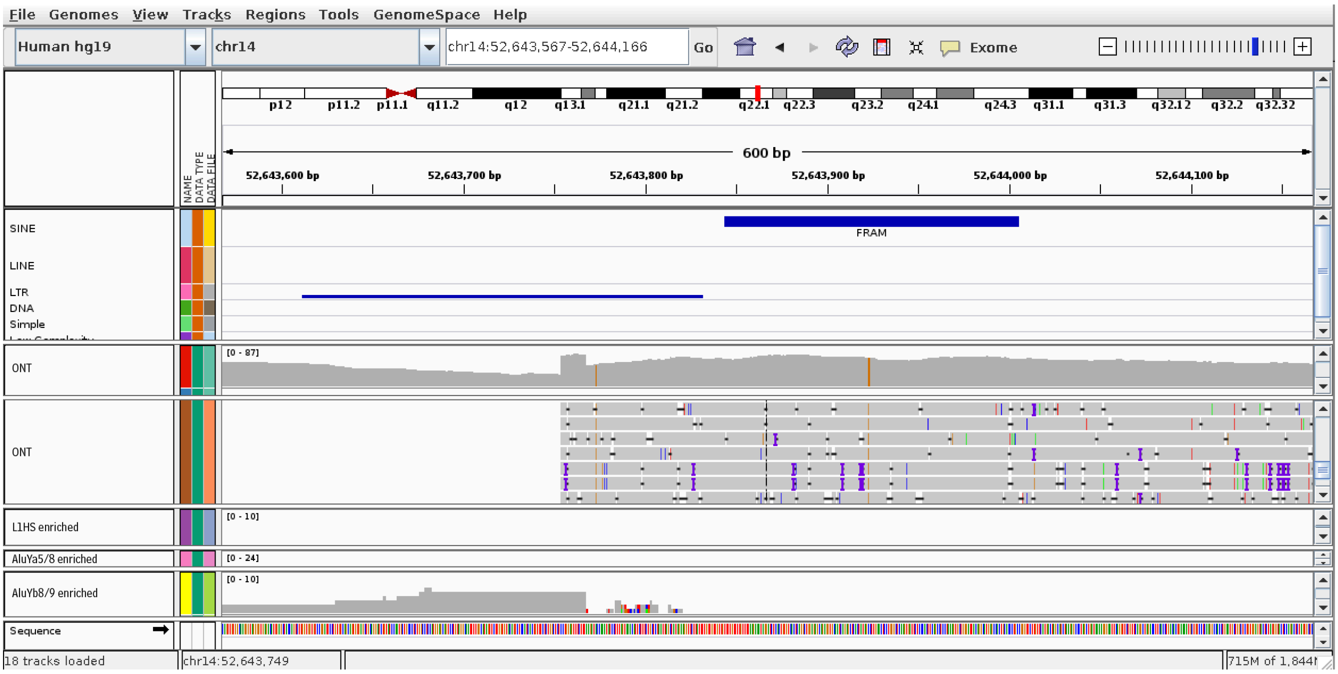
File Genomes View Tracks Regions Tools GenomeSpace Help
Human hg19
chr14
chr14:52,643,567-52,644,166
Go
Exome
600 bp
52,643,600 bp
52,643,700 bp
52,643,800 bp
52,643,900 bp
52,644,000 bp
52,644,100 bp
SINE
LINE
LTR
DNA
Simple
FRAM
ONT
[0 - 87]
ONT
L1HS enriched
[0 - 10]
AluYa5/8 enriched
[0 - 24]
AluYb8/9 enriched
[0 - 10]
Sequence
18 tracks loaded
chr14:52,643,749

B.

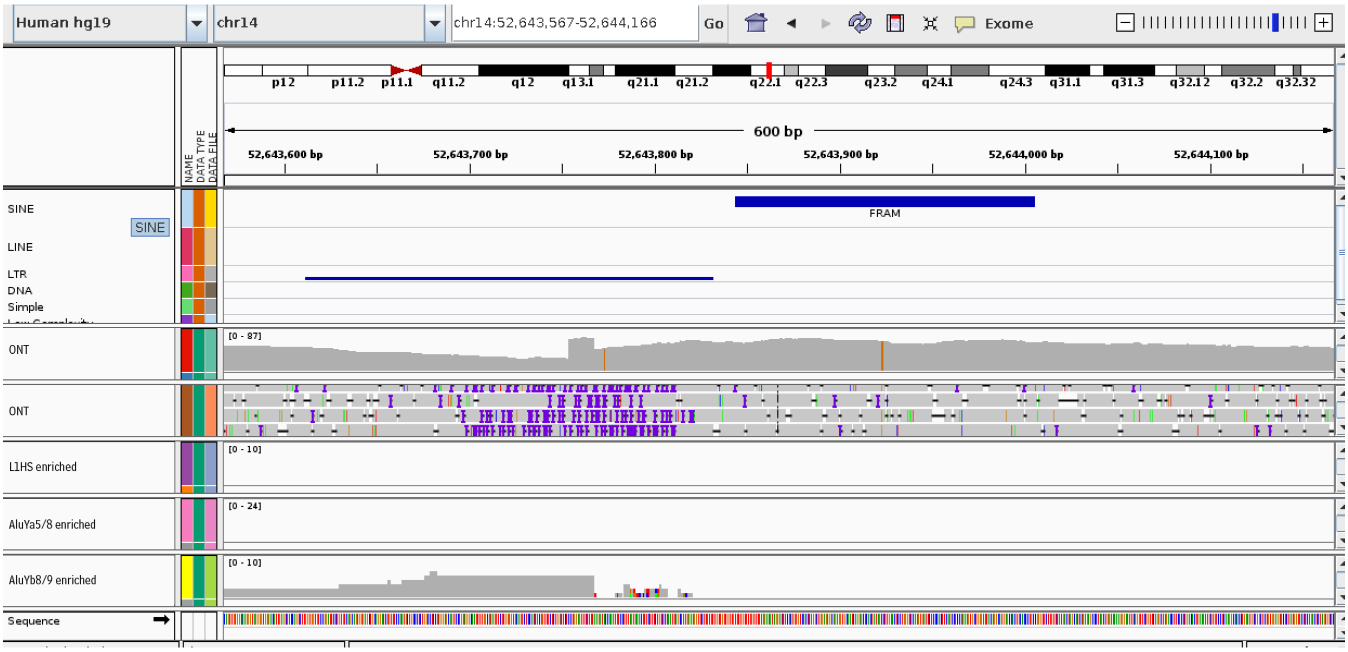
Human hg19
chr14
chr14:52,643,567-52,644,166
Go
Exome
600 bp
52,643,600 bp
52,643,700 bp
52,643,800 bp
52,643,900 bp
52,644,000 bp
52,644,100 bp
SINE
LINE
LTR
DNA
Simple
FRAM
ONT
[0 - 87]
ONT
L1HS enriched
[0 - 10]
AluYa5/8 enriched
[0 - 24]
AluYb8/9 enriched
[0 - 10]
Sequence

Fig. S5

A.

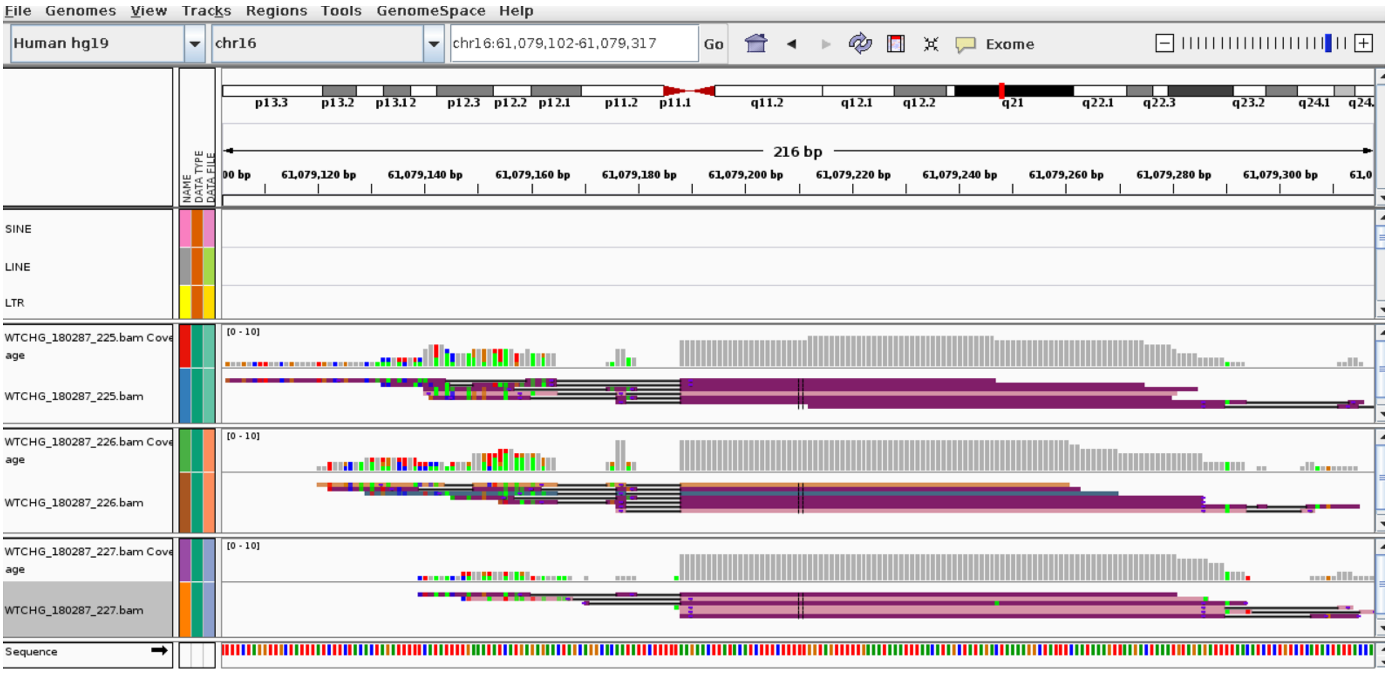
File Genomes View Tracks Regions Tools GenomeSpace Help
Human hg19
chr16
chr16:61,079,102-61,079,317
Go
Exome
216 bp
SINE
LINE
LTR
WTCHG_180287_225.bam Coverage
WTCHG_180287_225.bam
WTCHG_180287_226.bam Coverage
WTCHG_180287_226.bam
WTCHG_180287_227.bam Coverage
WTCHG_180287_227.bam
Sequence

B.

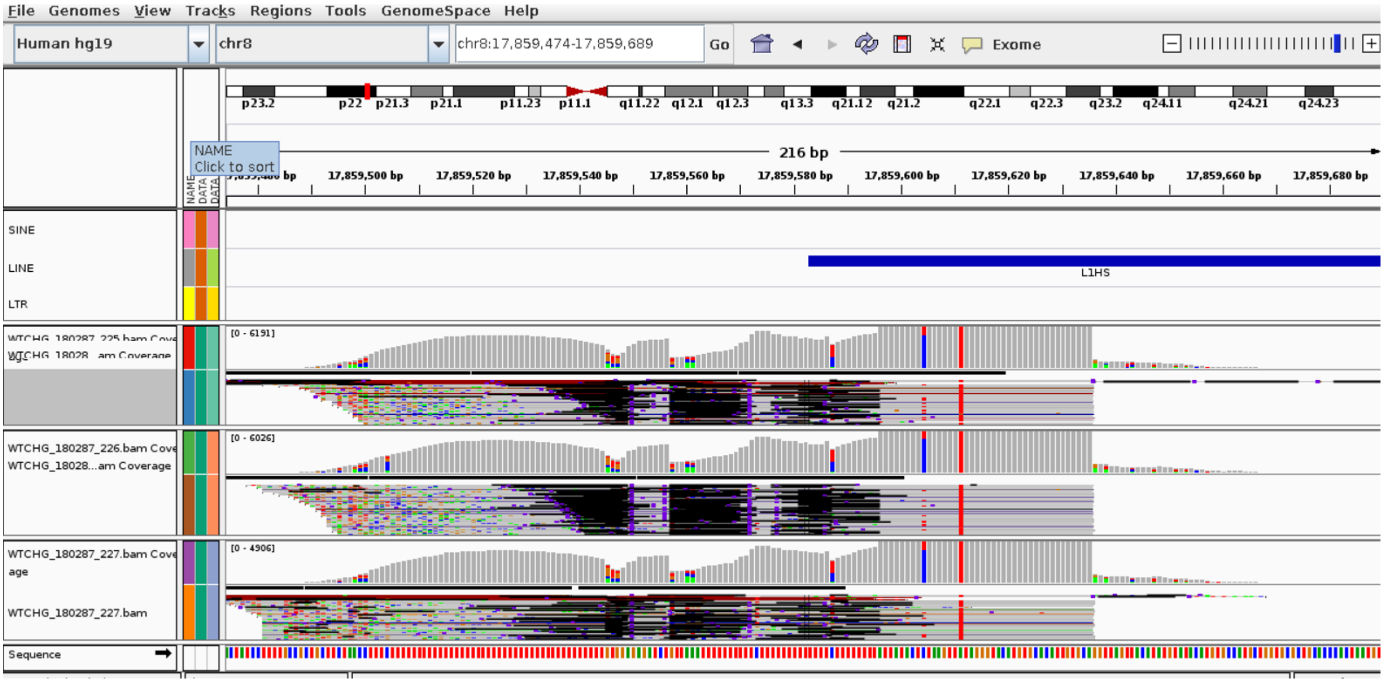
File Genomes View Tracks Regions Tools GenomeSpace Help
Human hg19
chr8
chr8:17,859,474-17,859,689
Go
Exome
216 bp
NAME
Click to sort
SINE
LINE
LTR
L1HS
WTCHG_180287_226.bam Coverage
WTCHG_180287_227.bam Coverage
WTCHG_180287_227.bam
Sequence

Fig. S6

A.

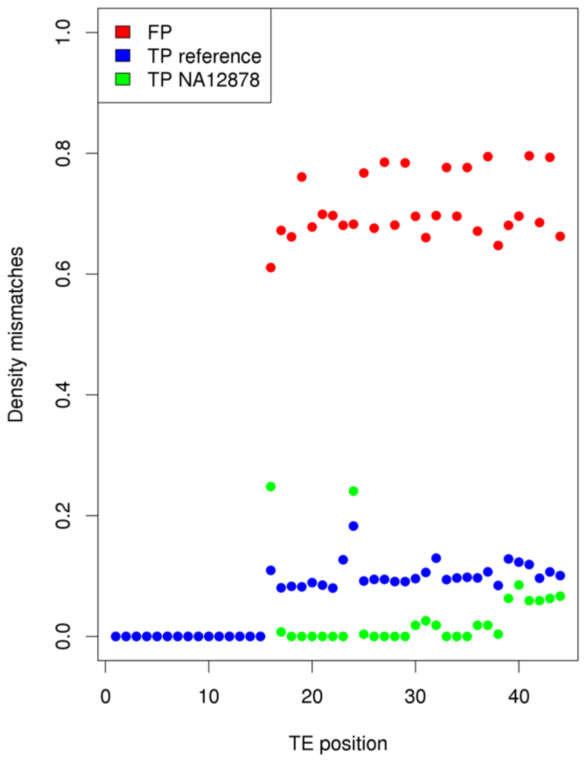

FP
TP reference
TP NA12878
Density mismatches
TE position
0.0
0.2
0.4
0.6
0.8
1.0
0
10
20
30
40

B.

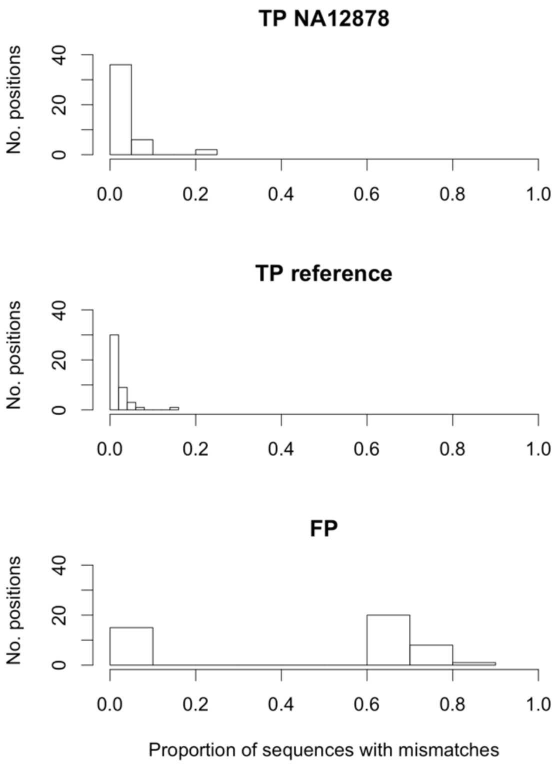

TP NA12878
TP reference
FP
No. positions
Proportion of sequences with mismatches
0
20
40
0.0
0.2
0.4
0.6
0.8
1.0

SUPPLEMENTARY REFERENCES

1. Stewart C, Kural D, Stromberg MP, Walker JA, Konkel MK, Stutz AM, Urban AE, Grubert F, Lam HY, Lee WP *et al*: **A comprehensive map of mobile element insertion polymorphisms in humans**. *PLoS Genet* 2011, **7**(8):e1002236.
2. Sudmant PH, Rausch T, Gardner EJ, Handsaker RE, Abyzov A, Huddleston J, Zhang Y, Ye K, Jun G, Hsi-Yang Fritz M *et al*: **An integrated map of structural variation in 2,504 human genomes**. *Nature* 2015, **526**(7571):75-81.
3. Ewing AD, Kazazian HH, Jr.: **Whole-genome resequencing allows detection of many rare LINE-1 insertion alleles in humans**. *Genome Res* 2011, **21**(6):985-990.
4. Illumina: **Oligonucleotide sequences © Illumina, Inc. All rights reserved.** In.; 2016.
5. Ewing AD, Kazazian HH, Jr.: **High-throughput sequencing reveals extensive variation in human-specific L1 content in individual human genomes**. *Genome Res* 2010, **20**(9):1262-1270.
